# Actions of N-arachidonyl-glycine in a rat inflammatory pain model

**DOI:** 10.1186/1744-8069-3-24

**Published:** 2007-08-30

**Authors:** Rebecca Succar, Vanessa A Mitchell, Christopher W Vaughan

**Affiliations:** 1 Pain Management Research Institute, Northern Clinical School, The University of Sydney at Royal North Shore Hospital, St Leonards, 2065, NSW, Australia

## Abstract

**Background:**

While cannabinoid receptor agonists have analgesic activity in inflammatory pain states they produce a range of side effects. Recently, it has been demonstrated that the arachidonic acid-amino acid conjugate, N-arachidonyl-glycine (NA-glycine) is effective in acute pain models.

**Results:**

In the present study we examined the effect of NA-glycine in a rat model of inflammatory pain. Intrathecal administration of NA-glycine (70 – 700 nmol) and the pan-cannabinoid receptor agonist HU-210 (10 nmol) reduced the mechanical allodynia and thermal hyperalgesia induced by intraplantar injection of Freund's complete adjuvant (FCA). The actions of HU-210, but not NA-glycine were reduced by the cannabinoid CB_1 _receptor antagonist AM251. The cannabinoid CB_2 _receptor antagonist SR144528 also had no effect on the actions of NA-glycine. In contrast, N-arachidonyl-GABA (NA-GABA, 700 nmol) and N-arachidonyl-alanine (NA-alanine, 700 nmol) had no effect on allodynia and hyperalgesia. HU-210, but not NA-glycine produced a reduction in rotarod latency.

**Conclusion:**

These findings suggest that NA-glycine may provide a novel non-cannabinoid receptor mediated approach to alleviate inflammatory pain.

## Background

The psychoactive ingredient of *Cannabis sativa*, Δ^9^-tetrahydrocannabinol (THC), is known to produce its physiological actions via an endogenous cannabinoid signalling system, specifically G-protein coupled cannabinoid CB_1 _and CB_2 _receptors [1]. There is now considerable evidence demonstrating that THC and a number of synthetic cannabinoid receptor agonists act via both cannabinoid CB_1 _and CB_2 _receptors to reduce the allodynia (pain due to normally non-noxious stimuli) and hyperalgesia (increased pain sensitivity to noxious stimuli) associated with inflammatory pain in animals [2-6]. However, non-selective cannabinoid agonists produce a spectrum of motor and psychotropic side effects, which are mediated by cannabinoid CB_1 _receptors [7-11].

Cannabinoid receptors are activated by endogenous cannabinoids (endocannabinoids), such as arachidonoyl ethanolamide (anandamide) and 2-arachidonyl glycerol. More recently, a number of arachidonyl-amino acid conjugates have been identified. One of these, N-arachidonyl glycine (NA-glycine), is expressed within the central nervous system, at particularly high levels within the spinal cord [12,13]. It has been proposed that NA-glycine is formed via oxidation of anandamide and by conjugation of glycine with arachidonic acid by arachidonyl-CoA [12,13]. NA-glycine differs from the endocannabinoid anandamide because it displays poor affinity for cannabinoid CB_1 _receptors [14]. Animal studies have shown that NA-glycine produces analgesia in acute pain models, reduces the allodynia associated with nerve ligation induced neuropathic pain and has anti-inflammatory activity [12,13,15,16]. In the present study we examined the effects of spinal administration of NA-glycine in an animal model of inflammatory pain.

## Results

Experiments were carried out in animals which had undergone chronic lumbar intrathecal catheter implantation. The mechanical paw withdrawal threshold, thermal paw withdrawal latency and rotarod latency were 14.9 ± 0.1 g, 13.3 ± 1.0 s and 151 ± 18 s prior to, and, 14.9 ± 0.1 g, 13.1 ± 0.7 s and 170 ± 25 s 3 days after intrathecal catheter implantation (n = 18). In one group of animals which did not receive intraplantar FCA, intrathecal injection of vehicle produced no significant change in mechanical paw withdrawal threshold, thermal paw withdrawal latency, or rotarod latency (P = 0.3 – 0.5 1-way ANOVA, n = 6).

### Effect of NA-glycine on mechanical paw withdrawal threshold

**Figure 1:**
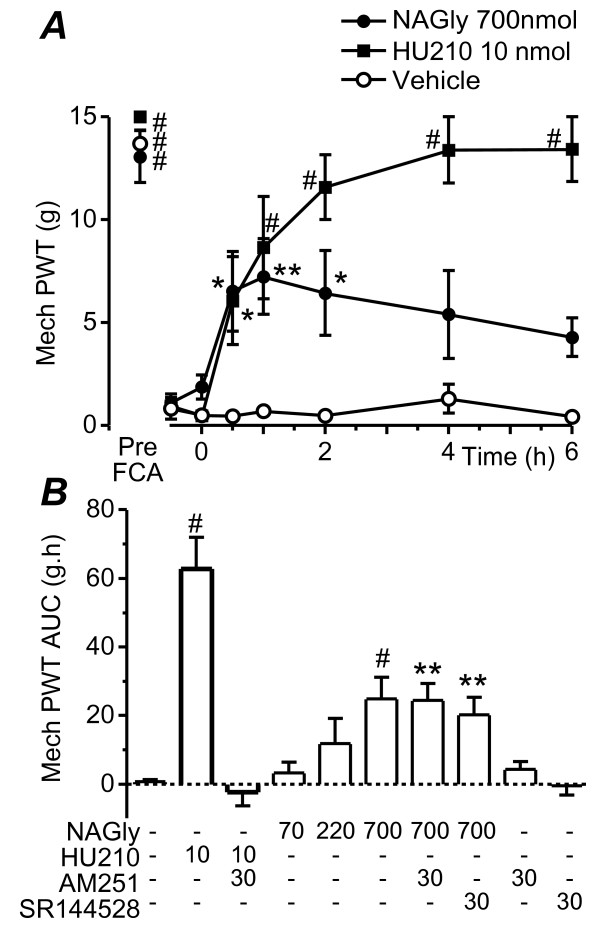
**NA-glycine reduces mechanical allodynia**. (a) Time plots of the effect of NA-glycine (NAGly,700 nmol, filled circles), HU-210 (10 nmol, filled squares), or vehicle (open circles) on mechanical paw withdrawal threshold (Mech PWT). Animals received an intrathecal injection of NA-glycine, HU-210 or a matched vehicle at time 0 h, 24 h after intraplantar injection of FCA. Data is also shown prior to FCA injection (Pre-FCA). (b) Bar charts depicting the effect of intrathecal injection of combinations of NA-glycine (NAGly 70 – 700 nmol), HU-210 (10 nmol), AM251 (30 nmol) and vehicle on mean mechanical paw withdrawal threshold, measured as the area-under-the-curve (AUC). * denotes P < 0.05, ** P < 0.01 and # P < 0.0001 compared to time 0 post-FCA in (a) and to vehicle in (b).

We first examined the time course of action of intrathecal administration of NA-glycine and the pan-cannabinoid receptor agonist HU-210 on mechanical paw withdrawal threshold at 24 h after intraplantar injection of FCA. In these animals, mechanical paw withdrawal threshold varied significantly over time (F_7,189 _= 65.4, P < 0.0001) and this differed between treatment groups (F_14,189 _= 12.0, P < 0.0001). Intraplantar injection of FCA produced a significant decrease in mechanical paw withdrawal threshold (Figure 1A, P < 0.0001). Intrathecal administration of NA-glycine (700 nmol) produced an increase in mechanical paw withdrawal threshold which peaked at 1 h and then declined over the 6 h time course (Figure 1A, n = 8). The increase in mechanical paw withdrawal threshold was significant at 0.5 – 2 h (P = 0.003 – 0.01) and was similar to the pre-inflammation level at 1 h (P = 0.06). Intrathecal administration of HU-210 (10 nmol) produced an increase in mechanical paw withdrawal threshold which was maintained over the 6 h time course (Figure 1A, n = 6). The increase in mechanical paw withdrawal threshold was significant at 1 – 6 h (P = 0.007 at 1 h, P < 0.0001 at 2 – 6 h) and was similar to the pre-inflammation level at 1 – 6 h (P = 0.1 at 1 h, P = 1.0 at 2 – 6 h). Intrathecal administration of vehicle did not produce a significant change in mechanical paw withdrawal threshold (Figure 1A; P = 1.0, n = 16).

We then assessed the effects of the NA-glycine and HU-210 on the mean mechanical paw withdrawal threshold, measured as the AUC. NA-glycine (700 nmol) produced an increase in the mean mechanical paw withdrawal threshold in the absence (n = 8) and presence of AM251 (30 nmol, n = 7), or SR144528 (30 nmol, n = 5) which was significantly greater than that produced by vehicle (n = 16) (Figure 1B, vehicle versus NA-glycine, P < 0.001; vehicle versus NA-glycine+AM251, P = 0.007; vehicle versus NA-glycine+SR144528, P = 0.001). The increase in mean mechanical paw withdrawal threshold was dose dependent, with smaller non-significant effects at 70 and 220 nmol doses (Figure 1B, P = 0.9, 0.2, n = 4, 6). HU-210 (10 nmol) produced an increase in the mean mechanical paw withdrawal threshold in the absence (n = 6), but not in the presence of AM251 (30 nmol, n = 4), which was significantly greater than that produced by vehicle (Figure 1B, vehicle versus HU-210, P < 0.0001; vehicle versus HU-210+AM251, P = 0.9). When administered alone AM251 (30 nmol, P = 0.9, n = 5) and SR144528 (30 nmol, P = 0.9, n = 4) had no significant effect on the mean mechanical paw withdrawal threshold (Figure 1B).

### Effect of NA-glycine on thermal paw withdrawal latency

**Figure 2:**
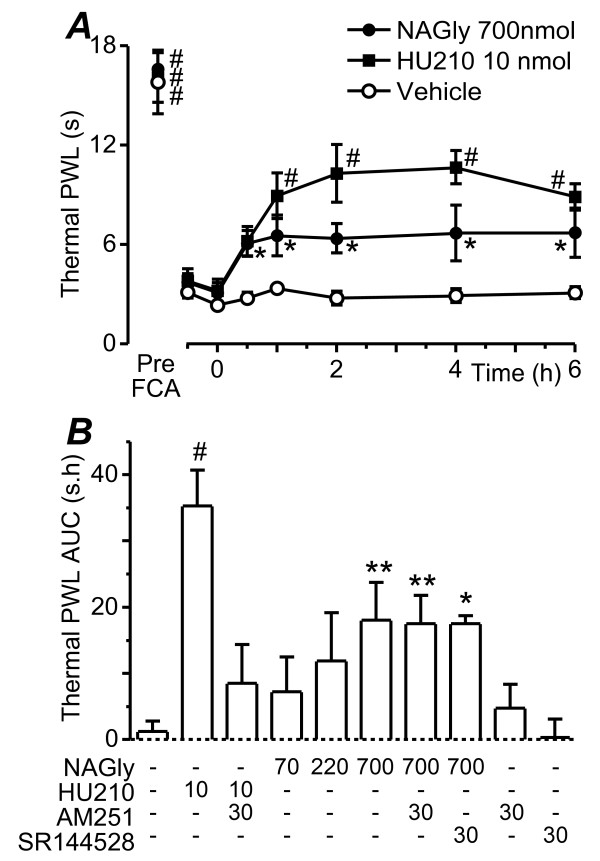
**NA-glycine reduces thermal hyperalgesia**. (a) Time plots of the effect of NA-glycine (NAGly,700 nmol, filled circles), HU-210 (10 nmol, filled squares), or vehicle (open circles) on thermal paw withdrawal latency (PWL). Animals received an intrathecal injection of NA-glycine, HU-210 or a matched vehicle at time 0 h, 24 h after intraplantar injection of FCA. Data is also shown prior to FCA injection (Pre-FCA). (b) Bar charts depicting the effect of intrathecal injection of combinations of NA-glycine (NAGly 70 – 700 nmol), HU-210 (10 nmol), AM251 (30 nmol) and vehicle on mean thermal paw withdrawal latency, measured as the area-under-the-curve (AUC). * denotes P < 0.05, ** P < 0.01 and # P < 0.0001 compared to time 0 post-FCA in (a) and vehicle in (b).

We next examined the time course of action of intrathecal NA-glycine and HU-210 on thermal paw withdrawal latency at 24 h after intraplantar injection of FCA. In these animals, thermal paw withdrawal latency varied significantly over time (F_7,189 _= 59.7, P < 0.0001) and this differed between treatment groups (F_14,189 _= 3.2, P = 0.0002). Intraplantar injection of FCA produced a significant decrease in thermal paw withdrawal latency (Figure 2A, P < 0.0001). Intrathecal administration of NA-glycine (700 nmol) produced an increase in thermal paw withdrawal latency which peaked at 1 h and was maintained over the 6 h time course (Figure 2A, n = 8). The increase in thermal paw withdrawal latency was significant at 0.5 – 6 h (P = 0.02 – 0.04), but was less than the pre-inflammation level at all time points (P < 0.01). Intrathecal administration of HU-210 (10 nmol) produced an increase in thermal paw withdrawal latency which peaked at 2 – 4 h and was maintained over the 6 h time course (Figure 2A, n = 6). The increase in thermal paw withdrawal latency was significant at 0.5 – 6 h (P = 0.03 at 0.5 h, P < 0.001 at 1 – 6 h) and was similar to the pre-inflammation level at 1 – 4 h (P = 0.6 – 0.8). Intrathecal administration of vehicle did not produce a significant change in thermal paw withdrawal latency (Figure 2A; P = 0.9 – 1.0, n = 16).

NA-glycine (700 nmol) produced an increase in the mean thermal paw withdrawal latency in the absence (n = 8) and presence of AM251 (30 nmol, n = 7), or SR144528 (30 nmol, n = 5) which was significantly greater than that produced by vehicle (n = 16) (Figure 2B, vehicle versus NA-glycine, P = 0.004; vehicle versus NA-glycine+AM251, P = 0.009; vehicle versus NA-glycine+SR144528, P = 0.02). The increase in mean thermal paw withdrawal latency was dose dependent, with smaller non-significant effects at 70 and 220 nmol doses (Figure 2B, P = 0.7, 0.2, n = 4, 6). HU-210 (10 nmol) produced an increase in the mean thermal paw withdrawal latency in the absence (n = 6), but not in the presence of AM251 (30 nmol, n = 4), which was significantly greater than that produced by vehicle (Figure 2B, vehicle versus HU-210, P < 0.0001; vehicle versus HU-210+AM251, P = 0.5). When administered alone AM251 (30 nmol, P = 0.8, n = 5) and SR144528 (30 nmol, P = 0.9, n = 4) had no significant effect on the mean thermal paw withdrawal latency (Figure 2B).

### Effect of other arachidonyl-amino acid conjugates

We also examined the effect of two other N-arachidonyl-amino acid conjugates. NA-GABA (700 nmol, AUC = 7.9 ± 2.1 g.h, P = 0.5, n = 7) and NA-alanine (700 nmol, AUC = 7.2 ± 2.6 g.h, P = 0.6, n = 6) did not produce an increase in mechanical paw withdrawal threshold significantly greater than that produced by vehicle (AUC = 0.7 ± 0.7 g.h). Similarly, NA-GABA (700 nmol, AUC = 2.5 ± 3.9 s.h, P = 0.9, n = 7) and NA-alanine (700 nmol, AUC = 1.6 ± 3.2 s.h, P = 0.9, n = 6) did not produce an increase in thermal paw withdrawal latency which was significantly greater than that produced by vehicle (AUC = 1.2 ± 1.6 s.h).

### Effect of NA-glycine on rotarod latency

**Figure 3:**
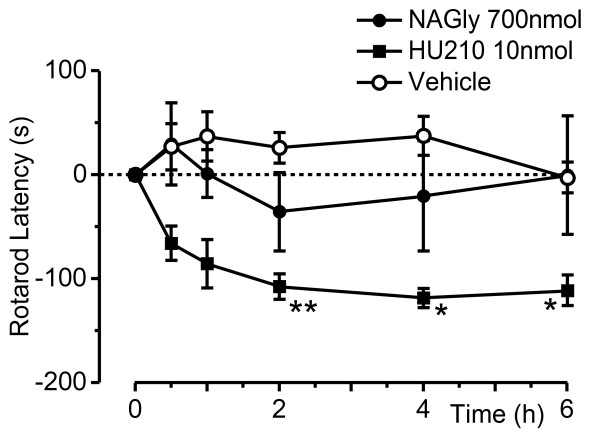
**NA-glycine does not affect motor function**. Time plots of the effect of NA-glycine (NAGly,700 nmol, filled circles), HU-210 (10 nmol, filled squares), or vehicle (open circles) on rotarod latency. Animals received an intrathecal injection of NA-glycine, HU-210 or a matched vehicle at time 0 h,24 h after intraplantar injection of FCA. Rotarod latency is shown as the difference to the latency at the time zero point, with negative values indicating a decrease in latency. * denotes P < 0.05, ** P < 0.01 and # P < 0.0001 compared to time 0.

We finally examined the time course of action of intrathecal NA-glycine and HU-210 on rotarod latency at 24 h after intraplantar injection of FCA. In these animals, rotarod latency varied significantly over time (F_5,85 _= 3.2, P = 0.01) and this differed between treatment groups (F_10,85 _= 3.1, P = 0.001). Intrathecal administration of NA-glycine (700 nmol) did not produce a significant change rotarod latency over the 6 h time course (Figure 3, P = 0.9 – 1.0, n = 5). Intrathecal administration of HU-210 (10 nmol) produced a decrease in rotarod latency which was significant at 2 – 6 h (Figure 3, P = 0.002 – 0.02, n = 6). Intrathecal administration of vehicle did not produce a significant change in rotarod latency over the 6 h time course (Figure 3; P = 0.8 – 1.0, n = 9).

## Discussion

The present study provides the first demonstration that the arachidonyl-amino acid conjugate NA-glycine reduces the allodynia and hyperalgesia associated with the FCA-induced model of inflammation in the rat, although to a lesser extent than the pan-cannabinoid receptor agonist HU-210. These observations are consistent with a recent study in which we demonstrated that spinally delivered NA-glycine also reduces the allodynia associated with a nerve-injury induced model of neuropathic pain [16]. In addition, previous studies have shown that NA-glycine produces analgesia following systemic administration in the hot plate test and following intraplantar administration in the formalin test [12,13]. The effects of NA-glycine were likely to be mediated by a specific protein because its actions were dose-dependent and were not reproduced by the related conjugates, NA-GABA and NA-alanine.

Like cannabinoid CB_1 _receptors, it has recently been demonstrated that cannabinoid CB_2 _receptors are expressed in the central nervous system [17] and may have mediated some of the effects observed in the present study. It has previously been demonstrated that systemically delivered non-selective cannabinoid receptor agonists reduce inflammatory pain via activation of both CB_1 _and CB_2 _receptors [2,4-6,11]. This is consistent with the observation that like cannabinoid CB_1 _receptors, CB_2 _receptors are expressed within spinal pain pathways and may have central analgesic actions, although there appears to some controversy as to whether this occurs in inflammatory, as opposed to neuropathic pain models [18-23]. The actions of intrathecal administration of HU-210 observed in the present study were likely to have been largely mediated by cannabinoid CB_1 _receptors because they were abolished by the cannabinoid CB_1 _receptor antagonist AM251. In contrast, the actions of intrathecal NA-glycine were not reduced by the cannabinoid CB_1 _and CB_2 _receptor antagonists, AM251 and SR144528. This is consistent with a recent study in which we found that AM251 and SR144528 did not affect the reduction in nerve injury induced allodynia produced by intrathecal NA-glycine [16]. This is also consistent with the moderate affinity of NA-glycine for the cannabinoid CB_1 _receptor [14], although NA-glycine's affinity for cannabinoid CB_2 _receptor remains to be determined.

NA-glycine may have produced its effects via other endocannabinoid related targets. NA-glycine is a substrate for the enzyme fatty acid amide hydrolase (FAAH), which is responsible for anandamide degradation [13,24]. It is possible that NA-glycine competition with anandamide for FAAH resulted in increased levels of anandamide, thereby producing cannabinoid receptor mediated anti-allodynia. This was unlikely to be responsible the observed effects because NA-glycine induced anti-allodynia was unaffected by cannabinoid receptor antagonists. The actions of NA-glycine were also unlikely to be due to activation of the vanilloid receptor TRPV1 because, unlike the structurally related endocannabinoid anandamide, NA-glycine does not activate TRPV1 [1,13]. It should also be noted NA-glycine is also a substrate for cyclooxygenase 2 (COX-2) [25] and a role for COX-2 in the observed effects cannot be excluded.

While the pharmacology of NA-glycine is still poorly understood, other targets for NA-glycine are emerging. NA-glycine is a ligand for the orphan receptor GPR18 [26] and has complex effects on prostaglandin synthesis [15]. In addition, it has recently been reported that NA-glycine inhibits the glycine transporter GLYT2, but has little effect on the glycine and GABA transporters, GLYT1 and GAT1 [27]. It is possible that at least part of the actions of NA-glycine were mediated by GLYT2 inhibition because GLYT2 is expressed at high levels within the spinal cord, and glycine receptor blockade produces allodynia in 'normal' animals and enhances nociceptive responses in pain pathways [28-30]. However, NA-alanine and NA-GABA, which also inhibit GLYT2a [27], had no significant effect in the present study. Thus, the mechanisms by which NA-glycine reduces inflammation induced allodynia and hyperalgesia remain to be determined.

Unlike HU-210, intrathecal NA-glycine had no effect on motor performance in the rotarod test. This is consistent with previous observations that intrathecal NA-glycine does not reduce rotarod performance in animals which have undergone partial sciatic nerve ligation [16] and that systemic administration of NA-glycine does not produce catalepsy in the ring test in normal animals [12]. The triad of cannabinoid CB_1 _receptor mediated side effects, including depression of spontaneous locomotor activity, catalepsy and hypothermia [e.g. [8]], was not examined in the present study, however, it has previously been shown that the rotarod test provides an indicator of cannabinoid CB_1 _receptor mediated side effects [10]. It should be noted that NA-glycine may produce motor side effects at higher doses, but this could not be examined because of solubility limitations. Equally, lower doses of intrathecal HU-210 may reduce allodynia without producing motor effects, as shown previously following systemic administration in a neuropathic pain model [9]. In this regard, the observed effects of NA-glycine were relatively small when compared to the synthetic cannabinoid HU-210, although they were of a similar potency and magnitude to those observed for NA-glycine in other pain models [13,16]. This relatively low potency may be due to a number of factors, such as enzymatic degradation which greatly reduces the effects of the structurally related endocannabinoid anandamide [31].

## Conclusion

Overall, the present study has demonstrated that NA-glycine may provide a useful tool for novel pain relieving strategies. The analgesic actions of NA-glycine may be complemented by a lack of motor side effects which are associated with THC and synthetic non-selective cannabinoid receptor agonists. Further studies are required to identify the targets of NA-glycine and more potent and efficacious ligands for these targets.

## Methods

### Animals

Male Sprague-Dawley rats, initially weighing between 160 and 200 g, were used for all experiments which were carried out following the guidelines of the NH&MRC 'Australian Code of Practice for the Care and Use of Animals for Scientific Purposes' (7^th ^Edition, 2004) and with the approval of the Royal North Shore Hospital/University of Technology Sydney Animal Care and Ethics Committee. Animals were housed in groups of three, under a 12:12 h light/dark cycle, with environmental enrichment and free access to food and water.

The following surgical procedures were carried out under isoflurane (1 – 3% in O_2_) anaesthesia. Chronic polyethylene lumbar intrathecal catheters were inserted between vertebrae L5–6, advanced 3 cm rostrally and exteriorised via the occipital region. Intrathecal injections of all agents were made in gently restrained animals via the exteriorised catheter (20 μl of agent, followed by 15 μl flush of saline to allow for dead-space in the catheter). For the inflammatory pain model, 0.15 ml of Freund's Complete Adjuvant (FCA, Sigma, Sydney, Australia) was injected subcutaneously into the plantar surface of the left hand paw.

### Behavioural measures and protocol

All behavioural testing was carried out in the light cycle. To assess mechanical allodynia, mechanical paw withdrawal thresholds were measured with a series of von Frey hairs (range 0.4 – 15 g). Rats were placed in elevated Perspex enclosures (28 cm × 15 cm × 18 cm) with wire mesh bases and given 15 – 20 min to acclimate to the testing environment. Each von Frey hair was tested 6 times at random locations on the plantar surface of the left hindpaw. Von Frey hairs were pressed perpendicularly against the hindpaw and held for approximately 2 s. Testing began with the 2.0 g von Frey hair. A positive withdrawal response was noted if the paw was sharply withdrawn, if any paw licking took place, or if the animal flinched upon removal of the von Frey filament. If the animal responded then the next lighter hair was tested. If the animal did not respond then the next heavier hair was tested. Once there was a change in response, four more hairs were tested and the mechanical paw withdrawal threshold was calculated using the up-down paradigm [32]. If the animals did, or did not respond to all hairs then the mechanical paw withdrawal threshold was assigned as 0.2 g, or 15 g, respectively.

To assess thermal hyperalgesia, thermal paw withdrawal latency of the left hand paw was measured using a plantar tester (Ugo Basile, Italy) [33]. Rats were placed in Perspex enclosures (15 × 15 × 18 cm) and given 10–15 min to acclimate. Focal infrared heat was applied through the plastic bottom of the cage to the left hind paw and the latency for the rat to respond by moving its paw away from the noxious heat source was recorded. To assess motor performance, the duration for which the animal could maintain balance on the rotating drum of a rotarod device (Ugo Basile, Italy) was measured as the rotarod latency, with a maximal cut-off time of 300 s.

All animals were allowed to acclimate to their holding cages for 3 days before any procedures were carried out. On day 3 animals were tested with all devices, including multiple training session on the rotarod. On day 4 animals were implanted with an intrathecal catheter. On days 5 and 6 animals were tested with all devices. On day 6, catheter placement was confirmed by the occurrence of bilateral hind limb paralysis following intrathecal injection of lignocaine hydrochloride (Sigma, Sydney, Australia, 2% dissolved in 0.9% saline). Any animals which did not display rapid, bilateral hind limb paralysis during the lignocaine test, or displayed abnormal gait following catheterisation were not used. On day 7, animals received an intraplantar injection of FCA. On day 8 animals underwent the experiment. Behavioural testing was carried twice out over a 60 min period before, then at set time points over a 6 h period following intrathecal drug injection. At the end of the experiment animals underwent another lignocaine test for catheter placement. Each animal underwent only one experiment.

### Drugs

N-[1-oxo-5Z,8Z,11Z,14Z-eicosatetraenyl]-glycine (N-arachidonyl glycine, NA-glycine), 4-[(1-oxo-5Z,8Z,11Z,14Z-eicosatetraenyl)amino-butanoic acid (N-arachidonyl GABA, NA-GABA), N-(1-oxo-5Z,8Z,11Z,14Z-eicosatetraenyl)-L-alanine (N-arachidonyl-alanine, NA-alanine) and 1-(2,4-dichlorophenyl)-5-(4-iodophenyl)-4-methyl-N-1-piperidinyl-1H–pyrazole-3-carboxamide (AM251) were obtained from Cayman Chemicals (Ann Arbor, USA) and SR144528 was a gift of Sanofi-Synthelabo (Mont Pellier, France). All drug combinations were made up in a vehicle solution comprising (V/V%) 6% ethanol and 2% Dimethyl sulfoxide in saline on the day of the experiment, and were injected intrathecally in a total volume of 20 μl, followed by a 15 μl flush of 0.9% saline.

### Data Analysis

In FCA-treated animals, statistical comparisons of each behavioural measure over time were made using a two-way analysis of variance (ANOVA), with time as a within-subjects factor and drug treatment as a between-subjects factor. Simple main effects for each behavioural measure were then tested for individual drug treatment group by comparing post-inflammation baseline values to post-intrathecal drug administration values and to pre-inflammation values, using Sidak's adjustment for multiple comparisons (except rotarod latency which was only compared to post-inflammation baseline values). Mean changes in allodynia and hyperalgesia produced by drug injection were calculated as the integral of post-injection values relative to pre-injection mean baseline (area-under-the-curve, AUC). Statistical comparisons of each behavioural AUC measure were made using a one-way ANOVA. When one-way ANOVAs were significant, post-hoc comparisons were made using Dunnett's adjustment for multiple comparisons to the vehicle group. In animals not treated with FCA statistical comparisons of each behavioural measure over time were made using a one-way ANOVA with time as a within-subjects factor. All data is presented as mean ± S.E.M.

## Competing interests

The author(s) declare that they have no competing interests.

## Authors' contributions

The study was conceived and the experiments were designed by CWV. RS and VAM performed the experiments and made contributions to the experimental design. RS, VAM and CWV all contributed to the analysis of the experiments and the preparation of the manuscript.
